# Cardioprotection of Ginkgolide B on Myocardial Ischemia/Reperfusion-Induced Inflammatory Injury via Regulation of A20-NF-κB Pathway

**DOI:** 10.3389/fimmu.2018.02844

**Published:** 2018-12-12

**Authors:** Rui Zhang, Lin Xu, Dong Zhang, Bo Hu, Qi Luo, Dan Han, Jiangbing Li, Chengwu Shen

**Affiliations:** ^1^Department of Pharmacy, Shandong Provincial Hospital Affiliated to Shandong University, Jinan, China; ^2^Department of Thoracic Surgery, Shandong Provincial Hospital Affiliated to Shandong University, Jinan, China; ^3^Department of Urology, Shandong Provincial Hospital Affiliated to Shandong University, Jinan, China; ^4^Minimally Invasive Urology Center, Shandong Provincial Hospital Affiliated to Shandong University, Jinan, China; ^5^Department of Pharmacy, Nanjing Drum Tower Hospital, The Affiliated Hospital of Nanjing University Medical School, Nanjing, China; ^6^Department of Cardiology, Shandong Provincial Hospital Affiliated to Shandong University, Jinan, China

**Keywords:** Ginkgolide B, Myocardial ischemia/reperfusion injury, Inflammation, A20, NF-κB

## Abstract

Inflammation urges most of the characteristics of plaques involved in the pathogenesis of myocardial ischemia/reperfusion injury (MI/RI). In addition, inflammatory signaling pathways not only mediate the properties of plaques that precipitate ischemia/reperfusion (I/R) but also influence the clinical consequences of the post-infarction remodeling and heart failure. Here, we studied whether Ginkgolide B (GB), an effective anti-inflammatory monomer, improved MI/RI via suppression of inflammation. Left anterior descending (LAD) coronary artery induced ischemia/reperfusion (I/R) of rats or A20 silencing mice, as well as hypoxia/reoxygenation (H/R) induced damages of primary cultured rat neonatal ventricular myocytes or A20 silencing ventricular myocytes, respectively, served as MI/RI model *in vivo* and *in vitro* to discuss the anti-I/R injury properties of GB. We found that GB significantly alleviated the symptoms of MI/RI evidently by reducing infarct size, preventing ultrastructural changes of myocardium, depressing Polymorphonuclears (PMNs) infiltration, lessening histopathological damage and suppressing the excessive inflammation. Further study demonstrated that GB remarkably inhibited NF-κB p65 subunit translocation, IκB-α phosphorylation, IKK-β activity, as well as the downstream inflammatory cytokines and proteins expressions via zinc finger protein A20. In conclusion, GB could alleviate MI/RI-induced inflammatory response through A20-NF-κB signal pathway, which may give us new insights into the preventive strategies for MI/RI disease.

## Introduction

Myocardial ischemia/reperfusion injury (MI/RI) with high morbidity and mortality rates has become one of the decisive factors for the events of cardiovascular diseases (1, 2). The mechanisms of MI/RI refer to a series of complicated pathological processes, including inflammatory response, calcium overload, complement activation, cell autophagy, and apoptosis (3). And, it has repeatedly been shown that the earliest phases of ischemia/reperfusion (I/R) are dominated by an acute inflammatory response. Presently, the mechanisms driving this acute and robust inflammatory response are still unknown. However, over the last decades, it has become increasingly clear that Zinc finger protein A20 is considered to be a pivotal link to the inflammation throughout the whole pathological process of myocardial ischemia/reperfusion induced tissue injury (4).

Zinc finger protein A20, also described as the TNF-α-induced protein 3 (TNFAIP3), is a widely expressed cytoplasmic signaling protein, commonly deemed as an anti-inflammatory, nuclear factor-kappa B (NF-κB) inhibitory, and anti-apoptotic molecule (5, 6). A20 was one key part of the mechanisms involved in multiple autoimmune and inflammatory diseases, such as coronary artery disease, psoriasis, systemic sclerosis, coeliac disease, type 1 diabetes, inflammatory bowel disease, and rheumatoid arthritis. A20 comprehensively results in alterations to the signaling pathways leading to inflammatory changes, and in consequence, regulates the intensity and duration of signaling by several critical factors mainly dependent on NF-κB pathway (7). And, we have also reported that up-regulating A20 could protect blood brain barrier against ischemic stroke superimposed on systemic inflammatory challenges (8). However, no data have been published focused on the role of A20 in pathogenesis of MI/RI.

Moreover, an increasing body of evidence suggested that I/R could elevate the activation of NF-κB, whereas inflammatory response was inhibited after NF-κB deactivation, and cardiac function restored. IκB-α, regarded as an inhibitor, binds to NF-κB p65/p50 heterodimer in cytoplasm (9–11). Phosphorylation and subsequent degradation of IκB-α caused by IKK-β activation lead to the release of NF-κB and then translocation to nucleus. Ultimately, that will stimulate the production of various inflammatory cytokines, such as interleukin (IL)-1β, tumor necrosis factor (TNF)-α, IL-6 and cell adhesion molecules which acts directly or indirectly to depress cardiac function (12). Meanwhile, PMNs infiltration also remarkably influences the post-ischemic perfused myocardium and various metabolites into the myocardial cells as well. Therefore, suppressing PMNs infiltration and NF-κB activation can obviously alleviate MI/RI induced damages and consequently offer myocardial protection (13, 14).

**Figure 1:**
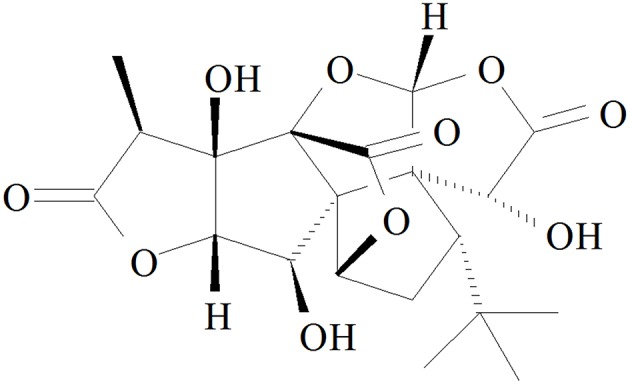
Chemical structure of GB.

Ginkgolide B (GB, Figure 1), an effective flavonoid monomer, was extracted from Ginkgo biloba leaves with multiple modulatory or protective functions and has been used in the treatment of cardio-cerebral vascular system damage for years (15–17). Most recently, researchers have discovered that GB could exert modulatory or protective functions against inflammatory reactions induced cascade effect to subsequently alleviate ischemia reperfusion diseases (18, 19). Moreover, there has been reported that GB could protect against IR-induced myocardial dysfunction and degradation of the membrane phospholipids (20). However, there has been no research reported on the relations between A20 and GB, meanwhile the specific mechanism of its anti-inflammatory effects is still limited and need an in-depth elucidation.

Thus, in this study, we investigated the role of GB in the protection of inflammation induced by MI/RI *in vivo*. We also made positive efforts to elucidate the role of A20-NF-κB signal pathway in the protection of ventricular myocytes exposed to H/R *in vitro*.

## Materials and Methods

### Materials and Reagents

GB (PubChem CID: 65243), 2, 3, 5-Triphenyltetrazolium chloride (TTC) was purchased from Sigma (St. Louis, MO, United States). DMEM medium (high glucose) and newborn calf serum were purchased from Gibco (Grand Island, NY, United States). TNF-α, IL-1β, and IL-6 ELISA kits were products of Sigma (St. Louis, MO, United States). Anti-A20, anti-ICAM-1, anti-VCAM-1, anti-iNOS, anti-NF-κB p65, anti-p-IκB-α, anti-IKK-β, anti-Histone, anti-β-actin, goat anti-rabbit and anti-mouse IgG antibodies were products of Santa Cruz (Santa Cruz, CA, United States). Enhanced chemiluminescence (ECL) plus kit was product of Keygen Biotech.

### Animals

Male Sprague-Dawley rats (250–300 g, Experimental Animal Center of Shandong University) were used for the current study. A20 gene silencing male mouse strains were provided by Beijing Biocytogen Co., Ltd. Rats and mice were housed in a temperature-controlled environment (18–22°C) with a 12 h light-dark cycle and allowed free access to food and water before the experiment. All the experiments were approved by the ethics committee of the Shandong Provincial Hospital affiliated to Shandong University (NSFC: No. 2018-019).

### *In vivo* I/R Procedure to Induce MI/RI in Rats

**Figure 2:**
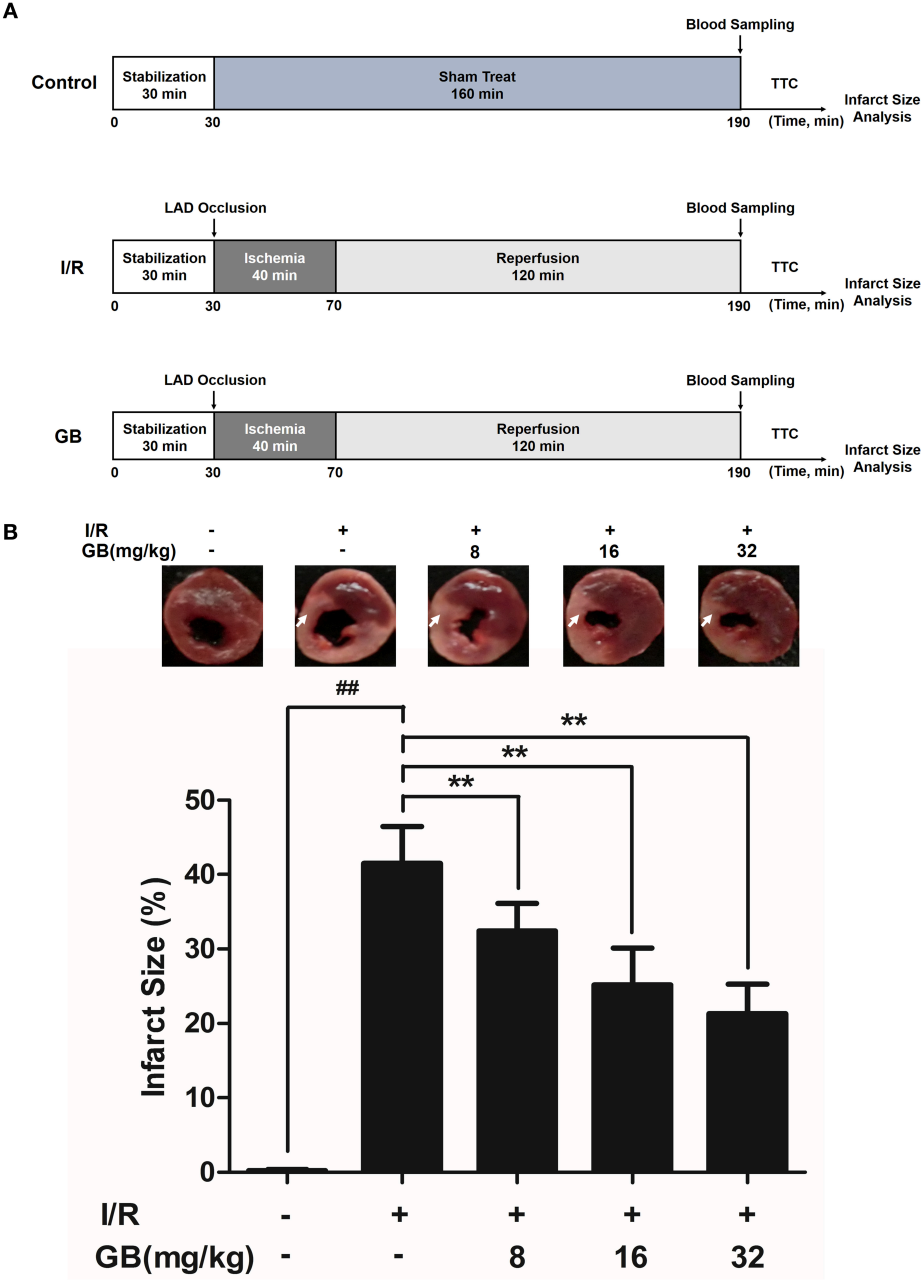
Effects of GB on infarct size in MI/RI rats model. **(A)** The experimental procedures of *in vivo* MI/RI rats model. **(B)** Treatment with GB significantly reduced the infarct size in MI/RI rats model. Data were expressed as mean ± S.D. (*n* = 8). ^##^*P* < 0.01, I/R group vs. control group; *P < 0.05, **P < 0.01, 8, 16, 32 mg/kg GB groups vs. I/R group.

I/R surgery was exactly carried out in accordance with the procedure in Figure 2A. The rats were anesthetized with 300 mg/kg chloral hydrate (i.p.). Electrocardiograph was continuously applied to monitor the changes of S-T segment so as to determine the success of surgery. After a left thoracotomy, the left anterior descending (LAD) coronary artery was twined with a plastic tube by a 6-0 silk suture for reversible LAD occlusion. Reperfusion for 120 min was initiated by releasing the suture and removing the tension after transient regional myocardial ischemia for 40 min according to the procedure. Before the rats were sacrificed, the blood samples were collected.

Randomly selected rats were divided into 5 groups as follows (*n* = 8 per group): (1) Control group, rats did not receive I/R, saline was administered; (2) I/R group, I/R rats administered with saline; (3) 8 mg/kg GB group, I/R rats received 8 mg/kg of GB; (4) 16 mg/kg GB group, I/R rats received 16 mg/kg of GB; (5) 32 mg/kg GB group, I/R rats received 32 mg/kg of GB. Saline and GB were, respectively administered intraperitoneally for 7 days before cardiac I/R operation.

### *In vivo* I/R Procedure to Induce MI/RI in A20 Gene Silencing Mice

I/R surgery was exactly carried out in accordance with the procedure in **Figure 5A**. The mice were anesthetized with 60 mg/kg 3% sodium pentobarbital (i.p.). Electrocardiograph was continuously applied to monitor the changes of S-T segment so as to determine the success of surgery. A longitudinal incision was made at the left margin of the sternum 2~3 mm and between the second and third costal points. The intercostal artery was ligatured and the thymus and pericardium were separated to expose the heart. After a left thoracotomy, the LAD coronary artery was tied in a slipknot using a 7-0 silk suture. In sham operated mice, silk sutures were placed around LAD but were not ligated. After transient regional myocardial ischemia for 30 min, unlock slipknot, the blood flow of the coronary artery was recovered for 90 min. Before the rats were sacrificed, the blood samples were collected.

Randomly selected mice were divided into 5 groups as follows (*n* = 8 per group): (1) Control group, A20 gene silencing mice did not receive I/R, saline was administered; (2) I/R group, I/R A20 gene silencing mice administered with saline; (3) 12 mg/kg GB group, I/R A20 gene silencing mice received 12 mg/kg of GB; (4) 24 mg/kg GB group, I/R A20 gene silencing mice received 24 mg/kg of GB; (5) 48 mg/kg GB group, I/R A20 gene silencing mice received 48 mg/kg of GB. Saline and GB were, respectively administered intraperitoneally for 7 days before cardiac I/R operation.

### Measurement of Infarct Size

Infarct size was determined by TTC staining technique. After I/R procedure, the hearts were excised and frozen at −80°C for 4 h. The left ventricle area of heart was cut into five 2–3 mm-thick slices from the apex to the base. After incubation in 2% TTC in PBS (pH 7.4) solution for 15 min (37°C), the third slice was immersed in formalin (4%) for another 30 min. Then, the area of the infarcted tissues was photographed with a digital camera and measured by Image-Pro Plus software (version 6.0, Media Cybernetic, United States) according to computerized planimetry. Infarct size was expressed as the percentage of infarcted area to the risk region × 100%.

### Transmission Electron Microscopy

The third heart slice was fixed in 3.0%, pH 7.2 glutaraldehyde buffered fixative for 2–3 days. Then the specimens were embedded in Polybed 812 before being rinsed in PBS. 60–80 nm-thick specimens were analyzed with a transmission electron microscope (JEM-2000EX) in three random fields.

### Histopathological Examination and Analysis of PMNS Infiltration Intensity

The third heart slice was stained with hematoxylin-eosin (H&E) and analyzed by light microscopy in three random fields. The intensity of histopathological damage was evaluated via pathological scores in accordance with the criteria reported by the previous study (21): (1) score 0: no damage; (2) score 1: mild damage; (3) score 2: moderate damage; (4) score 3: severe damage; (5) score 4: highly severe damage. The mean of the absolute number of PMNs was also recorded in three random high-power fields (HPF).

### Immunohistochemistry

Immunohistochemistry was applied to evaluate the expressions of ICAM-1, VCAM-1, and iNOS. The third heart slice was frozen and blocked by 10% normal serum. And, anti-ICAM-1, anti-VCAM-1 and anti-iNOS antibodies were incubated overnight at 4°C after being. Then, the heart slice was incubated with anti-rabbit IgG primary antibody for 30 min. Immunohisochemical staining protocol was used for further immunohistological analysis under the fluorescence microscope in three random fields. Image-Pro Plus software (version 6.0) was applied to quantify the optical density of positive staining area, as described previously (22). The results were expressed as mean optical density mean ± S.D.

### MPO Activity Assay

The ischemic tissue samples were homogenized and sonicated to release the MPO into the supernatant. Then, the activity of MPO was measured using kits according to the manufacture instructions (AmyJet Scientific Inc., Wuhan, China).

### *In vitro* H/R Procedure to Induce H/R Injury in Ventricular Myocytes

Rat ventricular myocytes were separated from the hearts of 1–4-day-old Sprague-Dawley rats according to trypsin enzymic digestion and differential attachment methods as described previously (21). Three days later, the cells were finally purified at a density of 1 × 10^5^/mL in DMEM medium supplemented with 10% fetal calf serum in 95% air/5% CO_2_ at 37°C.

H/R treatment procedure (**Figure 4A**). The cells were incubated in 95% N_2_/5% CO_2_ for 2 h (hypoxia) and then in 95% air/5% CO_2_ for 2 h (reoxygenation). Randomly selected cells were divided into five groups as follows (*n* = 8 per group): (1) Control group, cells did not receive H/R and were cultured in DMEM medium; (2) H/R group, cells receive H/R; (3) 1 μM GB group, H/R cells were pre-incubated with 1 μM GB for 24 h; (4) 10 μM GB group, H/R cells were pre-incubated with 10 μM GB for 24 h; (5) 100 μM GB group, H/R cells were pre-incubated with 100 μM GB for 24 h.

### Reconstruction of A20 Gene Silencing Ventricular Myocytes

After the density of ventricular myocytes reached 1 × 10^5^/mL, the cells were transfected with pGPU6/Hygro in control group while pGPU6/Hygro-A20 in other groups for 24 h using the GenePharma Transfection Reagent. After the transfection, cells were treated with H/R.

### *In vitro* H/R Procedure to Induce H/R injury in A20 Gene Silencing Ventricular Myocytes

The A20 gene silencing cells were incubated in 95% N_2_/5% CO_2_ for 2 h (hypoxia) and then in 95% air/5% CO_2_ for 2 h (reoxygenation). Randomly selected cells were divided into 5 groups as follows (*n* = 8 per group): (1) Control group, cells did not receive H/R and were cultured in DMEM medium; (2) H/R group, A20 gene silencing cells receive H/R; (3) 1 μM GB group, H/R A20 gene silencing cells were pre-incubated with 1 μM GB for 24 h; (4) 10 μM GB group, H/R A20 gene silencing cells were pre-incubated with 10 μM GB for 24 h; (5) 100 μM GB group, H/R A20 gene silencing cells were preincubated with 100 μM GB for 24 h.

### Analysis of Cell Vitality

Cell viability of ventricular myocytes was quantified with 3-(4,5-dimethylthiazol-2-yl)-2,5-diphenyl tetrazolium bromide (MTT) colorimetric assay. At the end of H/R procedure, cells were incubated with 5 mg/mL MTT for 4 h at 37°C and the insoluble formazan crystals were dissolved in 100 μl of DMSO for 15 min. Results were expressed as percentage of the optical density (OD) at 490 nm measured in control cells.

### Measurement of TNF-α, Il-1β, and Il-6

Before rats and mice were sacrificed, the blood samples were obtained. After H/R procedure, the cell supernatant was collected from medium. The expressions of TNF-α, IL-1β, and IL-6 were determined via ELISA kits both in blood samples and cell supernatant.

### Extraction of Myocardial Tissues Protein

RIPA Lysis Buffer (Beyotime Inc., China) and 1% phenylmethanesulfonyl fluoride (PMSF) were used to extract the proteins in myocardial tissue. Then, the myocardial tissues were homogenized and ultrasonically ground to no precipitation. Finally, the samples were centrifuged at 12,000 × g at 4°C for 30 min and the total protein was collected from the supernatant. All the steps above were carried out on the ice in order to avoid protein denaturation.

### Western Blot Analysis

Nuclear and Cytoplasmic Protein Extraction Kit (Beyotime Biotechnology, Beijing, China) was applied to extract the cytoplasmic and nuclear proteins from cells according to the manufacturer's instruction as described previously (21). The protein concentrations were determined by BCA assay.

Protein samples (50 μg) was loaded to SDS-PAGE gel, and then transferred to a PVDF membrane at 20 V and 100 mA overnight. The membranes were blocked with 5% skim milk, and then incubated with primary antibodies (1:800) against CD40, ICAM-1, VCAM-1, iNOS, NF-κB p65, p-IκB-α, and IKK-β proteins for 4 h at 37°C. The horseradish peroxidase-conjugated secondary antibody (1:1,000) was added and detected using an ECL plus kit. Protein expression levels were determined by quantitating protein band densities of images taken by Gel Imaging System using Quantity One software.

### Statistical Analysis

The results were expressed as the mean ± S.E.M. Significance of difference between groups were compared using one-way analysis of variance (ANOVA) followed by Bonferroni correction for multiple comparisons. A probability value of *P* < 0.05 was considered to be statistically significant. All statistical figures were performed using Graph Pad Prism software (Version 5.0).

## Results

### Effect of GB on MI/R-Induced Inflammatory Injury in MI/RI Rats Model

#### GB Reduced Infarct Size in MI/RI Rats

As the results shown in Figure 2B, infarct size in the I/R group was 41.5 ± 4.9% (*P* < 0.01 vs. control group), whereas 8, 16, 32 mg/kg GB decreased infarct size to 32.4 ± 3.7%, 25.2 ± 5.0% and 21.3 ± 4.0% (*P* < 0.01), respectively, compared with the I/R group.

#### GB Improved Cardiac Ultrastructural Characterization, Alleviated PMNS Infiltration, Decreased the Amount of Serum Inflammatory Cytokines and Inhibited Overexpressions of Myocardial Tissue ICAM-1, VCAM-1, and iNOS in MI/RI Rats

**Figure 3:**
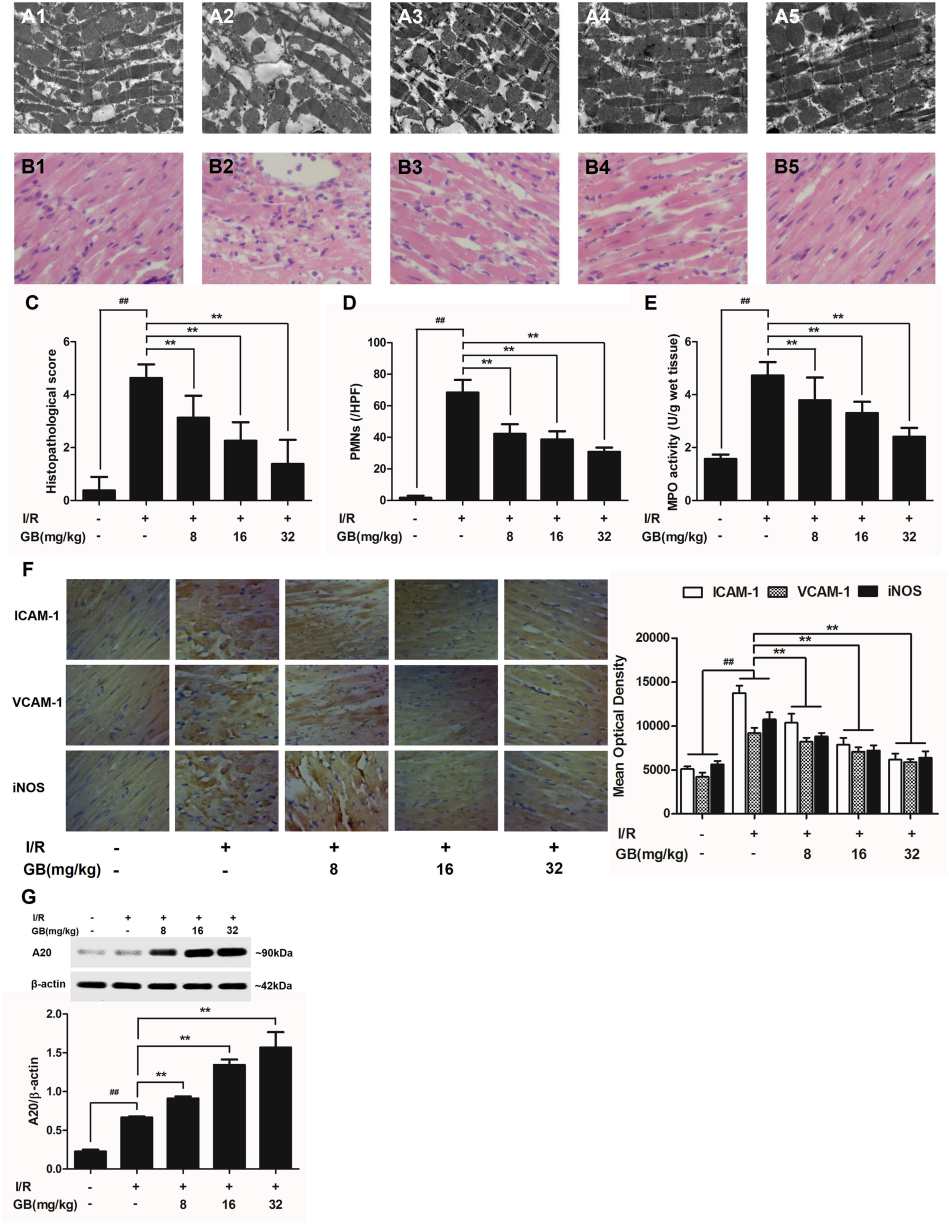
Effects of GB on the ultrastructure of myocardial tissue, histopathological changes, histopathological scores, PMNs counting, MPO activity and ICAM-1, VCAM-1, iNOS expressions in MI/RI rats model. **(A1–5)** Representative transmission electron microscopy (TEM) observation of myocardial tissue injury for control group **(A1)**, I/R group **(A2)**, I/R + 8 mg/kg GB group **(A3)**, I/R + 16 mg/kg GB group **(A4)**, I/R + 32 mg/kg GB group **(A5)**. **(B1-5)** Representative light microscopic appearance of rat myocardial histopathological morphology (HE staining; original magnification × 200) for control group **(B1)**, I/R group **(B2)**, I/R + 8 mg/kg GB group **(B3)**, I/R + 16 mg/kg GB group **(B4)**, I/R + 32 mg/kg GB group **(B5)**. **(C)** Effect of GB on histopathological scores, **(D)** effect of GB on myocardial PMNs counting, **(E)** effect of GB on MPO activity, effect of GB on expressions of ICAM-1, VCAM-1, iNOS **(F)** and effect of GB on expression of A20 **(G)**. The location of the histological images were taken in three random fields of infarcted area. Data were expressed as mean ± S.D. (*n* = 8). ^##^*P* < 0.01, I/R group vs. control group; *P < 0.05, **P < 0.01, 8, 16, 32 mg/kg GB groups vs. I/R group.

In the control group, mitochondria containing cristae with high electron density are elongated and tightly aligned between myofibrils (Figure 3A1). However, in I/R group, the cardiac myofibers were disconnected and damaged, nuclear stained deeper, and the mitochondria became swelling and degeneration (Figure 3A2). In 8 mg/kg GB group, there were still some breaks and loss of mitochondrial cristae associated with loss of the mitochondrial matrix (Figure 3A3). In 16 mg/kg GB group, the damaged mitochondria showed mild loss of cristae, swelling, myelin figures and membrane disruption (Figure 3A4). In 32 mg/kg GB group, only a few swollen mitochondria was observed (Figure 3A5).

In the control group, the myocytes arranged regularly and no inflammatory cells were observed in the myocardial interstitium. After I/R procedure, the myocytes arranged irregularly, the tissue became necrotic, and a large number of inflammatory cells were observed in the myocardial interstitium accompanied by the formation of fibrotic scars. But GB could significantly improve the histological injury, characterized by regularly arranged myocytes and alleviative inflammatory infiltration (Figures 3B1–5). As demonstrated in Figure 3C, 8, 16, 32 mg/kg GB markedly decrease the histopathological scores compared with I/R group (*P* < 0.01). Meanwhile, all GB groups remarkably decreased the total numbers of infiltrated and adherent PMNs compared with I/R group (Figure 3D).

Then, MPO activity was measured to evaluate the level of neutrophilic infiltration. In control group, the MPO activity was very low at 1.57 ± 0.16 U/g protein (Figure 3E). However, the MPO activity was significantly elevated in I/R group (4.72 ± 0.51 U/g protein) (*P* < 0.01 vs. control group). Interestingly, the current study indicated that pretreatment with GB 8 mg/kg (3.79 ± 0.86 U/g protein, *P* < 0.05), 16 mg/kg (3.30 ± 0.43 U/g protein, *P* < 0.01) and 32 mg/kg (2.40 ± 0.33 U/g protein, *P* < 0.01) could inhibit MPO activity in myocardial tissue compared with I/R group.

As shown in Table 1, the levels of TNF-α, IL-1β, and IL-6 were increased by 6.33-fold, 4.25-fold, and by 3.25-fold (*P* < 0.01), respectively, compared with control group. 8, 16, 32 mg/kg GB could dose-dependently decrease the levels of TNF-α by 23.2% (*P* < 0.05), 43.8% (*P* < 0.01) and 68.3% (*P* < 0.01), respectively, IL-1β by 22.5, 44.3, and 64.2% (*P* < 0.01), respectively, and IL-6 by 13.2% (*P* < 0.05), 39.9% (*P* < 0.01) and 51.3% (*P* < 0.01), respectively, compared with I/R group.

**Table 1:** Effects of GB on serum inflammatory cytokines in MI/RI rats model.

**Group**	**Dose (mg/kg)**	**TNF-α (pg/mL)**	**IL-1β (pg/mL)**	**IL-6 (pg/mL)**
Control		13.63 ± 3.61	56.41 ± 12.19	31.94 ± 8.34
I/R		86.34 ± 15.40^##^	239.56 ± 17.38^##^	103.78 ± 10.45^##^
I/R+GB	8	66.34 ± 10.98^**^	185.65 ± 21.76^**^	90.13 ± 14.53^*^
	16	48.54 ± 8.07^**^	133.49 ± 23.68^**^	62.34 ± 8.31^**^
	32	27.39 ± 6.31^**^	85.68 ± 9.78^**^	50.49 ± 3.98^**^

*Values were expressed as mean ± SD (n = 8)*.

## *P < 0.01 I/R group vs. control group*;

* *P < 0.05*,

** *P < 0.01, 8, 16, 32 mg/kg GB groups vs. I/R group*.

As shown in Figure 3F, the expressions of ICAM-1, VCAM-1, and iNOS in I/R group were significantly elevated compared with control group. However, 8, 16, 32 mg/kg GB could effectively reduce the expressions of ICAM-1, VCAM-1 and iNOS compared with I/R group. The original pictures were put in the Supplementary Material.

#### GB Increased Expression of A20 in MI/RI Rats

As shown in Figure 3G, the level of A20 in I/R group was obviously higher in contrast with control group (*P* < 0.01). But 8, 16, 32 mg/kg GB remarkably increased the level of A20 in response to I/R injury (*P* < 0.01 vs. I/R group).

### Effect of GB on H/R-Induced Injury in H/R Ventricular Myocytes Model

#### GB Increased Cell Viability Against H/R Injury in Ventricular Myocytes

**Figure 4:**
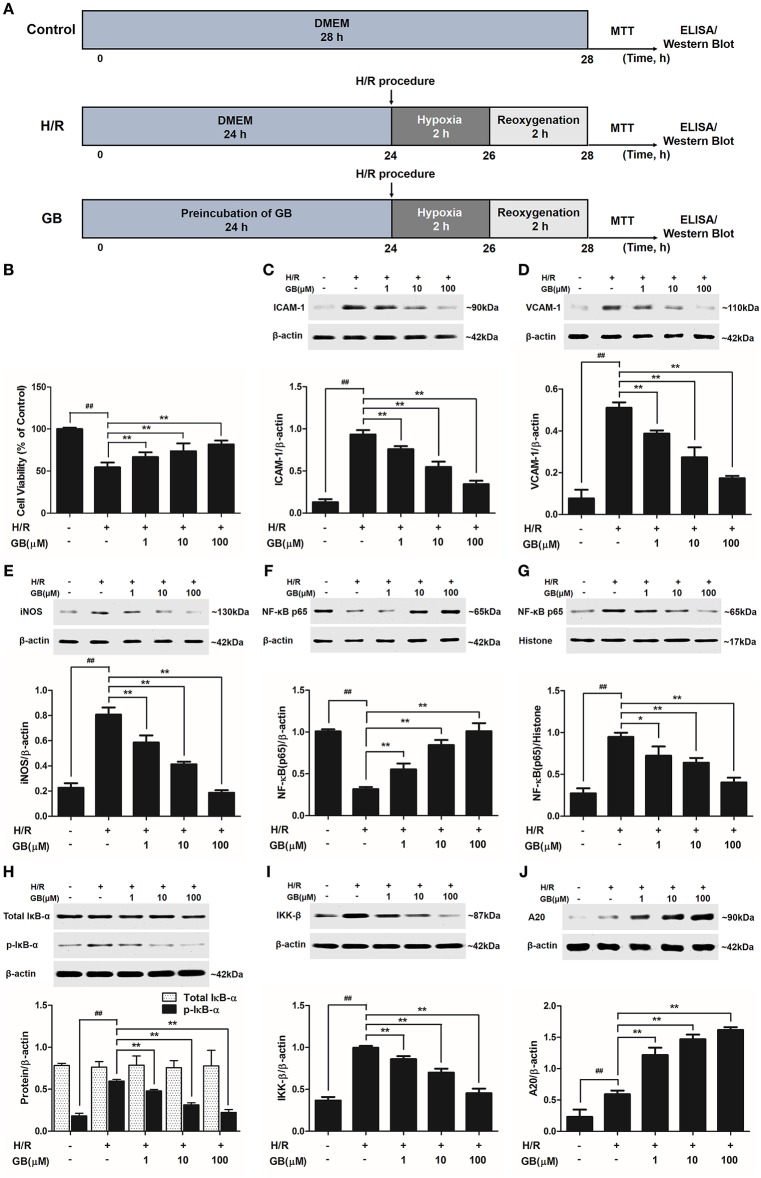
Effects of GB on cell viability and the expressions of ICAM-1, VCAM-1, iNOS, NF-κB p65, p-IκB-α, IKK-β by Western blot in H/R ventricular myocytes model. **(A)** The experimental procedures of *in vitro* H/R ventricular myocytes model. **(B)** GB significantly increased the cell viability after H/R procedure. **(C)** GB decreased the expression of ICAM-1. **(D)** GB decreased the expression of VCAM-1. **(E)** GB decreased the expression of iNOS. GB blocked the translocation of NF-κB p65 from cytosolic **(F)** to nuclear **(G)**. **(H)** GB down-regulated the expression of p-IκB-α. **(I)** GB decreased the expression of IKK-β. **(J)** GB increased the expression of A20. The NF-κB p65 protein levels were assayed separately in cytosolic **(F)** and nuclear **(G)** extracts. Results were expressed as Protein/reference protein ratio. Data were expressed as mean ± S.D. of three independent experiments. ^##^*P* < 0.01 H/R group vs. control group; *P < 0.05, **P < 0.01, 1, 10, 100 μM GB groups vs. I/R group.

As shown in Figure 4B, the cell viability in H/R group was markedly reduced to 54.5 ± 5.6% (*P* < 0.01 vs. control group). 1, 10, 100 μM GB could significantly increase the viability of cells received H/R injury (66.6 ± 5.8, 73.6 ± 9.3, 81.8 ± 4.7%, *P* < 0.01 vs. H/R group).

#### GB Inhibited Overproduction of Inflammatory Cytokines in H/R Ventricular Myocytes

The productions of TNF-α, IL-1β, and IL-6 in H/R group were markedly increased by 11.93-, 10.03-, and 29.50-fold, respectively, compared with control group (*P* < 0.01) in Table 2. Pretreatment with 1, 10, 100 μM GB could significantly reduce the levels of TNF-α by 30.4, 48.2, and 72.8% (*P* < 0.01), IL-1β by 39.5, 62.4, and 78.0% (*P* < 0.01), IL-6 by 26.4, 69.3, and 85.0% (*P* < 0.01), respectively, compared with H/R group.

**Table 2:** Effects of GB on supernatant inflammatory cytokines in H/R ventricular myocytes model.

**Group**	**Concentration (μM)**	**TNF-α (pg/mL)**	**IL-1β (pg/mL)**	**IL-6 (pg/mL)**
Control		5.21 ± 2.16	87.29 ± 6.89	23.10 ± 3.15
H/R		62.17 ± 5.96^##^	875.09 ± 47.10^##^	681.34 ± 29.32^##^
H/R+GB	1	43.26 ± 10.11^**^	529.21 ± 43.43^**^	501.21 ± 22.31^**^
	10	32.18 ± 2.98^**^	329.11 ± 20.98^**^	209.34 ± 13.34^**^
	100	16.88 ± 2.16^**^	192.10 ± 40.19^**^	102.09 ± 21.08^**^

*Values were expressed as mean ± SD (n = 8)*.

## *P < 0.01 H/R group vs. control group*;

^*^P < 0.05,

** *P < 0.01, 1, 10, 100 μM GB groups vs. I/R group*.

#### GB Prevented Overexpressions of ICAM-1, VCAM-1 and iNOS, Translocation of NF-κB p65, Phosphorylation of IκB-α, Activity of IKK-β and Increased Expression of A20 in H/R Ventricular Myocytes

Compared with control group, the expressions of ICAM-1, VCAM-1, and iNOS markedly increased to about 7.18-, 6.65-, and 3.56-fold (*P* < 0.01) after H/R procedure (Figures 4C–E). While pretreatment with 1, 10, 100 μM GB reduced the expressions of ICAM-1 by 18.6, 41.4, and 63.2% (*P* < 0.01), VCAM-1 by 24.2, 46.4, and 66.0% (*P* < 0.01) and iNOS by 27.3, 48.8, and 76.9% (*P* < 0.01) compared with H/R group.

As shown in Figures 4F,G, the levels of NF-κB p65 were relatively high in the cytoplasm of cells but low in nucleus in control group. However, an evident translocation of p65 from the cytosol into nucleus showed in H/R group. Pretreatment with 1, 10, 100 μM GB could inhibit such nuclear translocation in a concentration-dependent manner.

As shown in Figure 4H, the total IκB-α in each group was not different. Then we checked the level of p-IκB-α in each group. Compared with control group, the level of p-IκB-α in H/R group significantly increased by 3.25-fold (*P* < 0.01). However, 1, 10, 100 μM GB all showed a notably inhibitory effect on phosphorylation of IκB-α by 19.6, 47.5, and 62.6% (*P* < 0.01) compared with H/R group.

And, we furtherly checked whether GB had an influence on IKK-β activity. The results in Figure 4I showed that the level of IKK-β significantly increased by 2.72-fold in H/R group (*P* < 0.01 vs. control group). In contrast, 1, 10, 100 μM GB could reduce the expressions of IKK-β by 13.7, 29.8, and 54.5% (*P* < 0.01) compared with H/R group.

As shown in Figure 4J, there was a small increased expression of A20 after H/R procedure (*P* < 0.01 vs. control group). While pre-incubation of GB (1, 10, 100 μM) all significantly enhanced the expressions of A20 in response to H/R injury (*P* < 0.01 vs. H/R group).

### Effect of GB on MI/R-Induced Inflammatory Injury in A20 Gene Silencing MI/RI Mice Model

**Figure 5:**
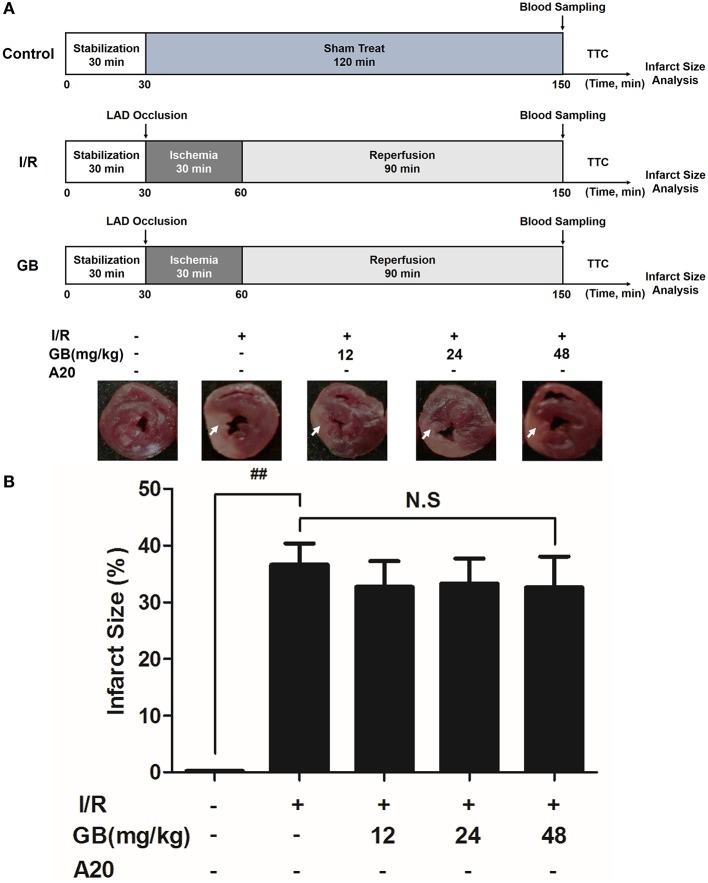
Effects of GB on infarct size in A20 gene silencing MI/RI mice model. **(A)** The experimental procedures of *in vivo* A20 gene silencing MI/RI mice model. **(B)** Treatment with GB significantly reduced the infarct size in A20 gene silencing MI/RI mice model. Data were expressed as mean ± S.D. (*n* = 8). ^##^*P* < 0.01, I/R group vs. control group; *P < 0.05, **P < 0.01, 12, 24, 48 mg/kg GB groups vs. I/R group.

**Figure 6:**
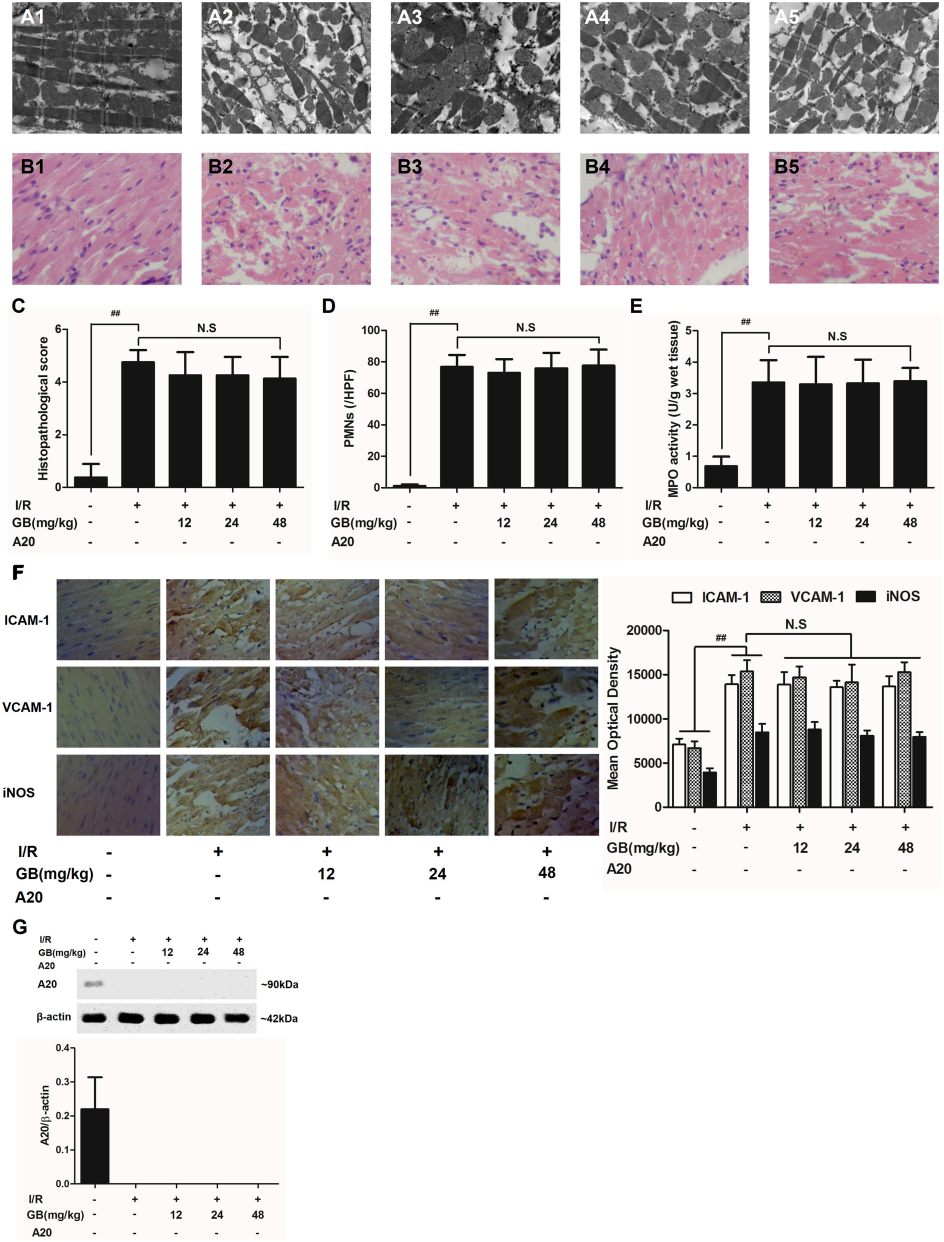
Effects of GB on the ultrastructure of myocardial tissue, histopathological changes, histopathological scores, PMNs counting, MPO activity and ICAM-1, VCAM-1, iNOS expressions in A20 gene silencing MI/RI mice model. **(A1-5)** Representative transmission electron microscopy (TEM) observation of myocardial tissue injury for control group **(A1)**, I/R group **(A2)**, I/R + 12 mg/kg GB group **(A3)**, I/R + 24 mg/kg GB group **(A4)**, I/R + 48 mg/kg GB group **(A5)**. **(B1-5)** Representative light microscopic appearance of rat myocardial histopathological morphology (HE staining; original magnification × 200) for control group **(B1)**, I/R group **(B2)**, I/R + 12 mg/kg GB group **(B3)**, I/R + 24 mg/kg GB group **(B4)**, I/R + 48 mg/kg GB group **(B5)**. **(C)** Effect of GB on histopathological scores, **(D)** effect of GB on myocardial PMNs counting, **(E)** effect of GB on MPO activity, effect of GB on expressions of ICAM-1, VCAM-1, iNOS **(F)** and effect of GB on expression of A20 **(G)**. The location of the histological images were taken in three random fields of infarcted area. Data were expressed as mean ± S.D. (*n* = 8). ^##^*P* < 0.01 I/R group vs. control group; *P < 0.05, **P < 0.01, 12, 24, 48 mg/kg GB groups vs. I/R group.

In I/R group, it was found that I/R procedure could markedly increase infarct size (Figure 5B), destroy cardiac ultrastructural characterization (Figures 6A1–5), aggravate pathological changes (Figures 6B1–5C), trigger PMNs infiltration (Figure 6D), cause inflammatory cytokines overproduction (Table 3) and upregulate expressions of ICAM-1, VCAM-1, iNOS (Figure 6F). As shown in Figure 6G, there showed successful and stable A20 gene silencing in mice except the control group. It was interesting that, after A20 gene was silenced, 12, 24, 48 mg/kg GB all failed to improve the outcomes induced by MI/RI (Figure 6 and Table 3).

**Table 3:** Effects of GB on serum inflammatory cytokines in A20 gene silencing MI/RI mice model.

**Group**	**Dose (mg/kg)**	**TNF-α (pg/mL)**	**IL-1β (pg/mL)**	**IL-6 (pg/mL)**
Control		34.21 ± 6.44	39.53 ± 10.98	18.39 ± 3.14
I/R		134.31 ± 10.98^##^	193.53 ± 23.67^##^	76.55 ± 6.98^##^
I/R+GB	12	119.82 ± 14.33^*^	153.12 ± 17.09^**^	68.05 ± 3.29^*^
	24	87.09 ± 5.09^**^	89.31 ± 14.13^**^	52.12 ± 6.99^**^
	48	40.01 ± 13.32^**^	58.39 ± 4.56^**^	48.15 ± 6.11^**^

*Values were expressed as mean ± SD (n = 8)*.

## *P < 0.01, I/R group vs. control group*;

* *P < 0.05*,

** *P < 0.01, 12, 24, 48 mg/kg GB groups vs. I/R group*.

### Effect of GB on H/R-Induced Injury in A20 Gene Silencing H/R Ventricular Myocytes Model

#### GB Could Not Increase Cell Viability After A20 Gene Silencing

**Figure 7:**
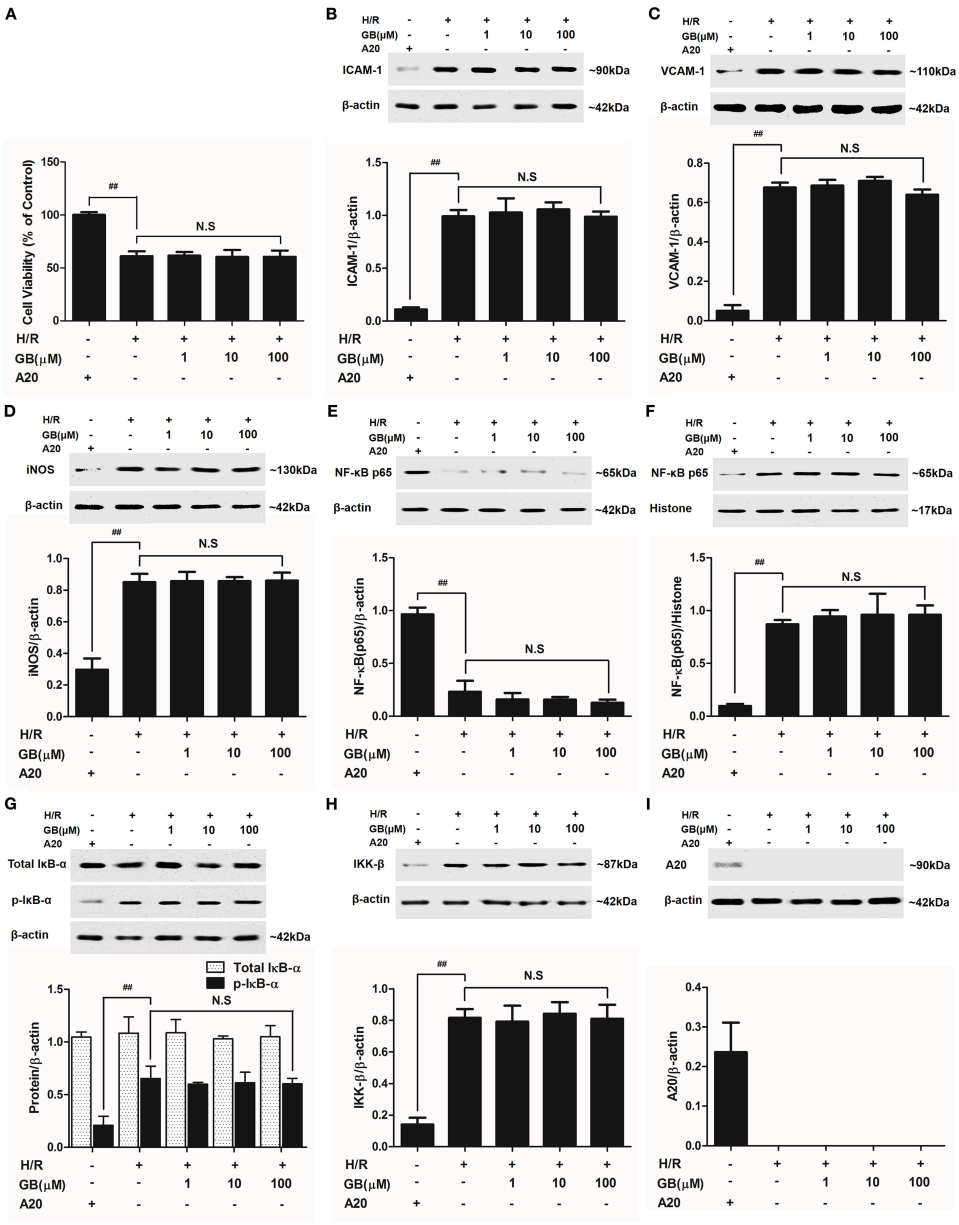
Effects of GB on cell viability **(A)** and the expressions of **(B)** ICAM-1, **(C)** VCAM-1, **(D)** iNOS, **(E)** cytoplasm NF-κB p65, **(F)** nucleus NF-κB p65, **(G)** p-IκB-α, **(H)** IKK-β and **(I)** A20 by Western blot in A20 gene silence H/R ventricular myocytes model. Results were expressed as Protein/reference protein ratio. Data were expressed as mean ± S.D. of three independent experiments. ^##^*P* < 0.01, H/R group vs. control group; *P < 0.05, **P < 0.01, 1, 10, 100 μM GB groups vs. I/R group.

The cell viabilities in H/R + A20 silencing group (Figure 7A) were significantly reduced (*P* < 0.01 vs. control group). After A20 gene silencing, GB could not elevate the cell viability against to H/R injury.

#### GB Could Not Inhibit the Expression of Inflammatory Factors, Translocation of NF-κB p65, Phosphorylation of IκB-α and Activity of IKK-β After A20 Gene Silencing

Compared with control group, the expressions of TNF-α, IL-1β, IL-6, ICAM-1, VCAM-1, and iNOS in H/R + A20 silencing group were obviously increased (Table 4 and Figures 7B–D). Whereas, after A20 gene silencing, GB could not influence the expressions of TNF-α, IL-1β, IL-6, ICAM-1, VCAM-1, and iNOS.

**Table 4:** Effects of GB on supernatant inflammatory cytokines in A20 gene silence H/R ventricular myocytes model.

**Group**	**Concentration (μM)**	**TNF-α (pg/mL)**	**IL-1β (pg/mL)**	**IL-6 (pg/mL)**
Control		4.13 ± 0.18	69.33 ± 18.25	16.19 ± 2.26
H/R+A20^−^		72.19 ± 6.59^##^	906.19 ± 73.20^##^	729.35 ± 81.00^##^
H/R+GB+A20^−^	1	68.33 ± 10.31	872.12 ± 67.34	681.98 ± 69.21
	10	62.18 ± 5.68	912.33 ± 89.76	633.10 ± 58.22
	100	65.33 ± 8.32	890.65 ± 76.38	637.39 ± 58.31

*Values were expressed as mean ± SD (n = 8)*.

## *P < 0.01, H/R group vs. control group*;

^*^P < 0.05, ^**^P < 0.01, 1, 10, 100 μM GB groups vs. I/R group.

The levels of NF-κB translocation, IκB-α phosphorylation and IKK-β activity were significantly affected in H/R + A20 silence group (Figures 7E–H). Nevertheless, after A20 gene silencing, all GB groups had no impact on NF-κB p65 translocation, IκB-α phosphorylation and IKK-β activity compared with H/R + A20 silence group.

#### Stable A20 Gene Silencing in Ventricular Myocytes

As shown in Figure 7I, there was little A20 expressed after transfection by preincubation with pGPU6/Hygro in control group. In addition, the ventricular myocytes in other groups were preincubated with pGPU6/Hygro-A20 and no A20 expressed after transfection.

## Discussion

This is the first investigation studied on MI/RI both *in vivo* and *in vitro* to examine whether GB played a vital role in the whole pathological process of MI/RI, whether GB regulated the expression of A20 in response to H/R injury, and whether NF-κB signal pathway was heavily involved in the whole pathogenesis.

Inflammation is responsible for the development of many cardiovascular or cerebrovascular diseases, such as atherosclerosis, myocardial infarction, stroke, hyperlipidemia, and neurodegeneration (23, 24). And, it is an important form of cardiomyocyte death in the early stage of MI/RI, which further leads to severe complications such as arrhythmia and heart failure. It was traditionally believed that the process of I/R gradually provokes severe inflammatory response and subsequent cardiac rupture, ventricular aneurysm formation, and exacerbation of left ventricular (LV) remodeling (25). However, not only the pathogenesis of I/R may lead to the inflammatory response, but inflammation itself may aggravate the I/R injury. But so far, the information of molecular mechanisms underlying the proinflammatory process of MI/RI is far from certain. Therefore, study on inflammation participating in MI/RI is quite meaningful for preventive therapy.

Recently, herbal treatment of cardiovascular and cerebrovascular diseases has gained much attention. GB, a major monomer of extracts from leaves of *Ginkgo biloba* traditionally used in Chinese herbal medicine, displays a wide range of biological activities, including anti-inflammatory and anti-oxidant effects (26). It has been reported that GB could exert neuroprotective effects against cerebral ischemia/reperfusion injury (CI/RI) via suppressing inflammatory response and scavenging oxygen free radical (27, 28). Both MI/RI and CI/RI are hypoxic and anoxic diseases, which have similar characteristics in pathogenesis and treatment, suggesting that GB may possess a potential value in the treatment strategy of MI/RI. Meanwhile, we have just proved that Ginkgolide C (GC) which possessed the similarity chemical structure to GB could exert a protective effect against MI/RI via inhibiting inflammation which might involve in blocking CD40-NF-κB signal pathway (21). However, it has not been illuminated whether GB has the similar effect of improving MI/RI yet. Consequently, in this study we investigated whether the strong anti-inflammatory property of GB constituted a part of molecular mechanisms of MI/RI protective effect.

It is well established that infarct size is a very important indicator reflecting therapeutic effect of MI/RI. One of the most effective strategy for reducing the size followed by acute myocardial infarction is the early and successful myocardial reperfusion which can improve the clinical outcome to a great degree (29). In this study, we found that pretreatment of GB for 7 days could remarkably protect against the I/R insult through significant reduction of infarct size. In addition, we found that GB largely improves myocardium damage as evidenced by restoration of myocardial ultrastructure and suppression of myofibrillar degeneration.

PMNs, which are involved in multiple non-infectious inflammatory processes including the response to MI/RI, serves as a key effector in the innate immune system (30). Following MI/RI, a large increase in the number of circulating PMNs occurred, which could predict the major adverse cardiac events in MI/RI patients with larger infarct size and worse cardiac function (31). Therefore, suppression of PMNs infiltration is a main resource of relieving the damage of MI/RI. In this study, a convincing show of histopathological damage was found in *in vivo* I/R groups which suggested that there was a definite relationship between MI/RI and PMNs infiltration. Nevertheless, pretreatment with GB could significantly attenuate PMNs infiltration, as determined by histopathological scores and the counting of PMNs. MPO, regarded as a precise marker of MI/RI and a risk factor for long-term mortality, is secreted by PMNs. Similarly, the elevated level of MPO in I/R groups was decreased after pretreatment with GB. These findings provide a potential link between GB's MI/RI protective effect and PMNs infiltration inhibitory property.

Moreover, *in vitro* H/R-treated ventricular myocytes were applied to imitate the pathological process of *in vivo* I/R injury, which helped to thoroughly validate the cardioprotective property of GB. Interestingly, we found for the first time that GB could significantly increase the cell viability after I/R-like insult which indicated that GB could exert anti-MI/RI effect via promoting viability and tolerance of cells injured by H/R-induced inflammation.

So far, observed data implicate that NF-κB is deemed as one of the key players in pathogenesis of I/R injury. And they have also shown that active NF-κB-mediated signaling significantly increased I/R-induced heart damage (32, 33). NF-κB functions as a crucial transcription factor in both inflammatory cells and myocardial cells, linking the coordinated inflammatory and cell death signaling pathways proposed in the concept of necroinflammation. However, lots of studies indicated that inhibition of NF-κB signal pathways could remarkably suppress the inflammation induced by MI/RI (34–36). Under normal conditions, NF-κB proteins were bound by members of the inhibitor of κB (IκB) family as components of inactive cytoplasmic complexes (37, 38). After ubiquitylation and proteasomal degradation of phosphorylated IκB family members, nuclear translocation of NF-κB family members would be released (39). In the present work, the level of NF-κB p65 translocation was obviously elevated after H/R procedure. Whereas, our results indicated that GB could effectively reverse this activated effect. In addition, pretreatment with GB could significantly block the notable phosphorylation of IκB-α triggered by H/R procedure. These results suggest that the reduction in phosphorylation of IκB-α and translocation of NF-κB p65 is at least one of the targets of GB for inhibiting I/R-induced inflammation.

Genetic evidence suggests that IKK complex (IKK-α, β, and γ), a supporting role in activating the NF-κB pathway, is pivotal for activating phosphorylation-dependent IκB degradation and NF-κB nuclear translocation (40). Therefore, we detected whether GB had an influence on the activity of IKK-β. Unsurprisingly, GB could also inhibit IKK-β activation in H/R injured ventricular myocytes. Consequently, we determined that IKK-β/IκB-α/NF-κB signal pathway was one of the anti-inflammatory targets of GB.

Numerous data have proved that there was a positive feedback between NF-κB activation and its downstream inflammatory cytokines, such as TNF-α, IL-1β, and IL-6; cell adhesion molecules, such as ICAM-1 and VCAM-1; and nitric oxide synthase (NOS) (41–43). Therefore, this study has also checked this point. It implies that, blockade of NF-κB pathway by GB has shown positive efficacy in the management of MI/RI induced inflammation.

**Figure 8:**
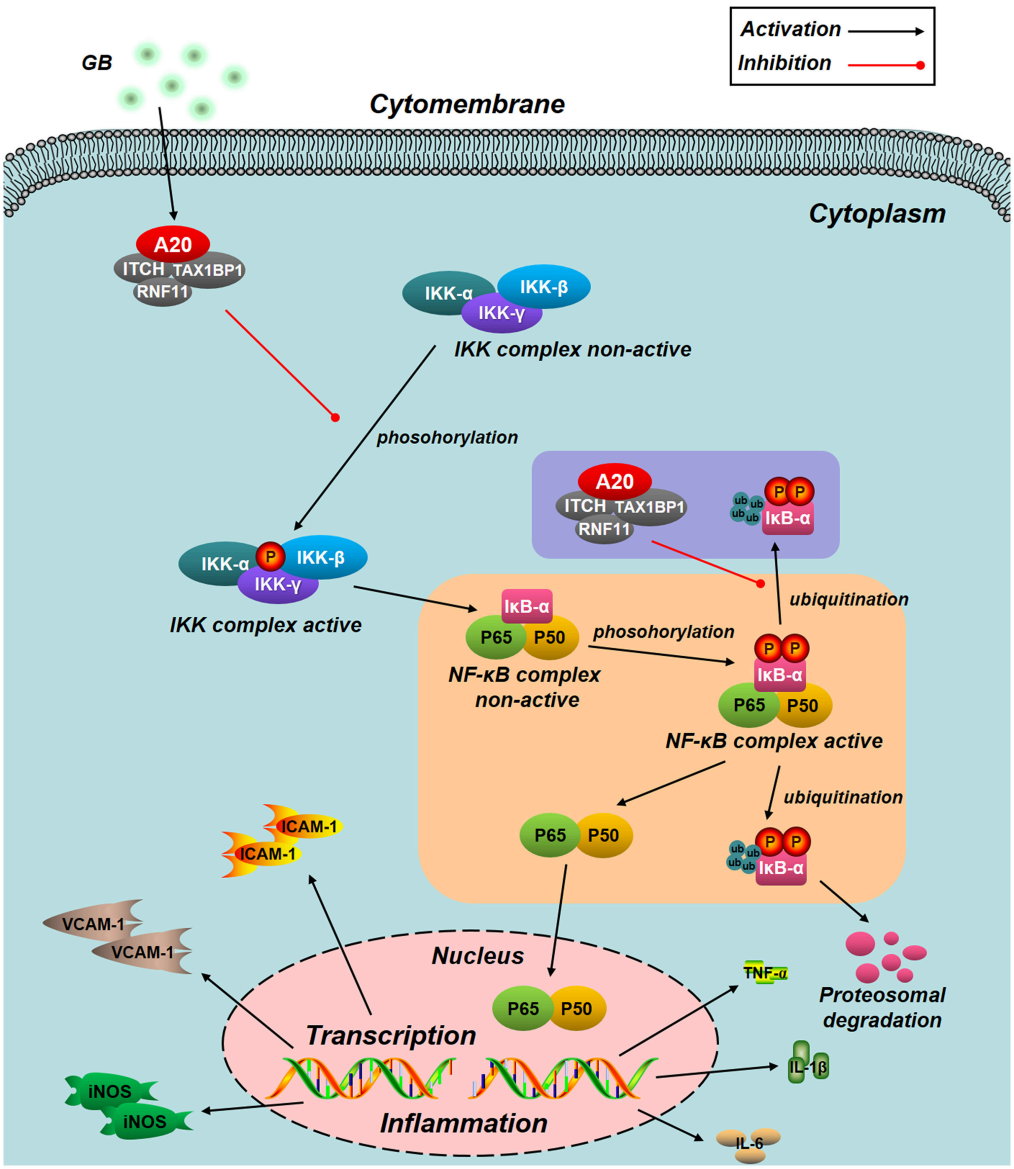
Schematic diagram describing the mechanism in the inhibitory effect of GB on H/R induced ventricular myocytes inflammatory injury. GB could alleviate MI/RI-induced inflammatory injury via up-regulating A20 and inhibiting IKK/IκB/NF-κB signal pathway.

NF-κB signal pathway is mediated by several regulatory mechanisms to keep the homeostasis of tissue. Zinc finger protein A20, serves as a tumor suppressor gene and susceptibility gene/biomarker of disease, involved in various inflammatory diseases, especially MI/RI (44, 45). Previous studies have verified that A20 was a central and inducible negative regulator of NF-κB which regulates multiple inflammatory signaling cascades. Silencing of A20 can significantly promote the translocation of NF-κB p65, finally leading to a pro-inflammatory state (46). Our present study showed that the level of A20 is low under normal conditions. However, A20 was significantly up-regulated by all GB-treated groups and the consequences were severe inhibition of NF-κB signal pathway. According to the fact, we concluded that the MI/RI protective effect of GB might partly attribute to NF-κB inhibition mediated by upregulation of A20. Furthermore, we silence the A20 gene both *in vivo* and *in vitro* to verify our hypothesis. After A20 was silenced, GB failed to reduce infarct size, improve cardiac ultrastructural characterization, inhibit PMNs infiltration and downregulate expressions of inflammatory cytokines and proteins *in vivo*. In addition, GB had no effect on cell viability and inflammatory factors at all. (Data Sheet 1 in Supplementary Material). Therefore, it was obvious that GB exerted the protective effect against MI/RI through inhibiting NF-κB signal pathway via A20 (Figure 8).

In conclusion, this is the first time to find out that GB alleviated MI/RI-induced inflammatory insult both *in vivo* and *in vitro* via up-regulating A20 dependent NF-κB signal pathway. Thus, GB could be applied as a preventive and valuable agent against MI/RI.

## Author Contributions

RZ, LX, DZ, BH, and QL performed the research. RZ, DH, JL, and CS designed the research study. RZ and CS analyzed the data. RZ wrote and edited the paper.

### Conflict of Interest Statement

The authors declare that the research was conducted in the absence of any commercial or financial relationships that could be construed as a potential conflict of interest.

## Supplementary Material

Supplementary Figure 1 The LPS experimental procedure *in vitro*.

Supplementary Figure 2 Effects of GB on cell viability and the expressions of A20, ICAM-1, VCAM-1, iNOS, NF-κB p65, p-IκB-α, IKK-β after LPS procedure.

Supplementary Figure 3 Effects of GB on cell viability and the expressions of A20, ICAM-1, VCAM-1, iNOS, NF-κB p65, p-IκB-α, IKK-β after A20 silencing.
