# Supplementary figures and images for: Cardioprotection of Ginkgolide B on Myocardial Ischemia/Reperfusion-Induced Inflammatory Injury via Regulation of A20-NF-κB Pathway
Source: Front Immunol. 2018 Dec 12;9:2844. doi: 10.3389/fimmu.2018.02844 (PMC6299132; doi:10.3389/fimmu.2018.02844)

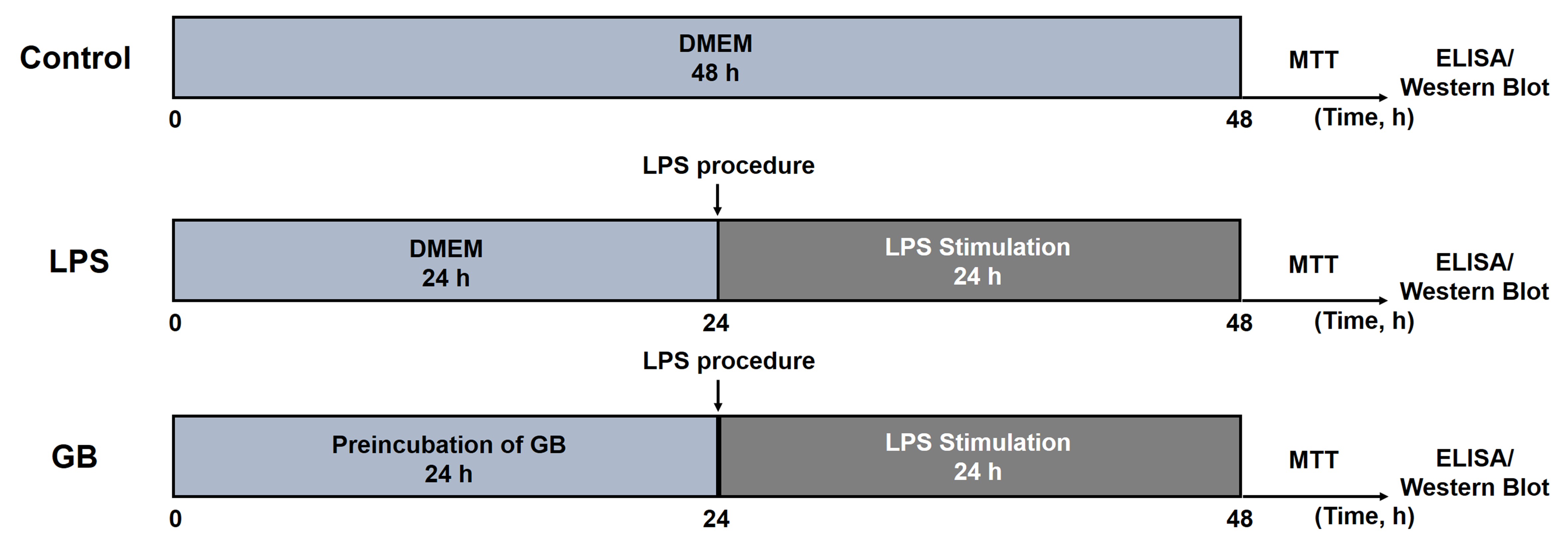

Supplement: Supplementary Figure 1 — The LPS experimental procedure in vitro. [file Image_1.tif]

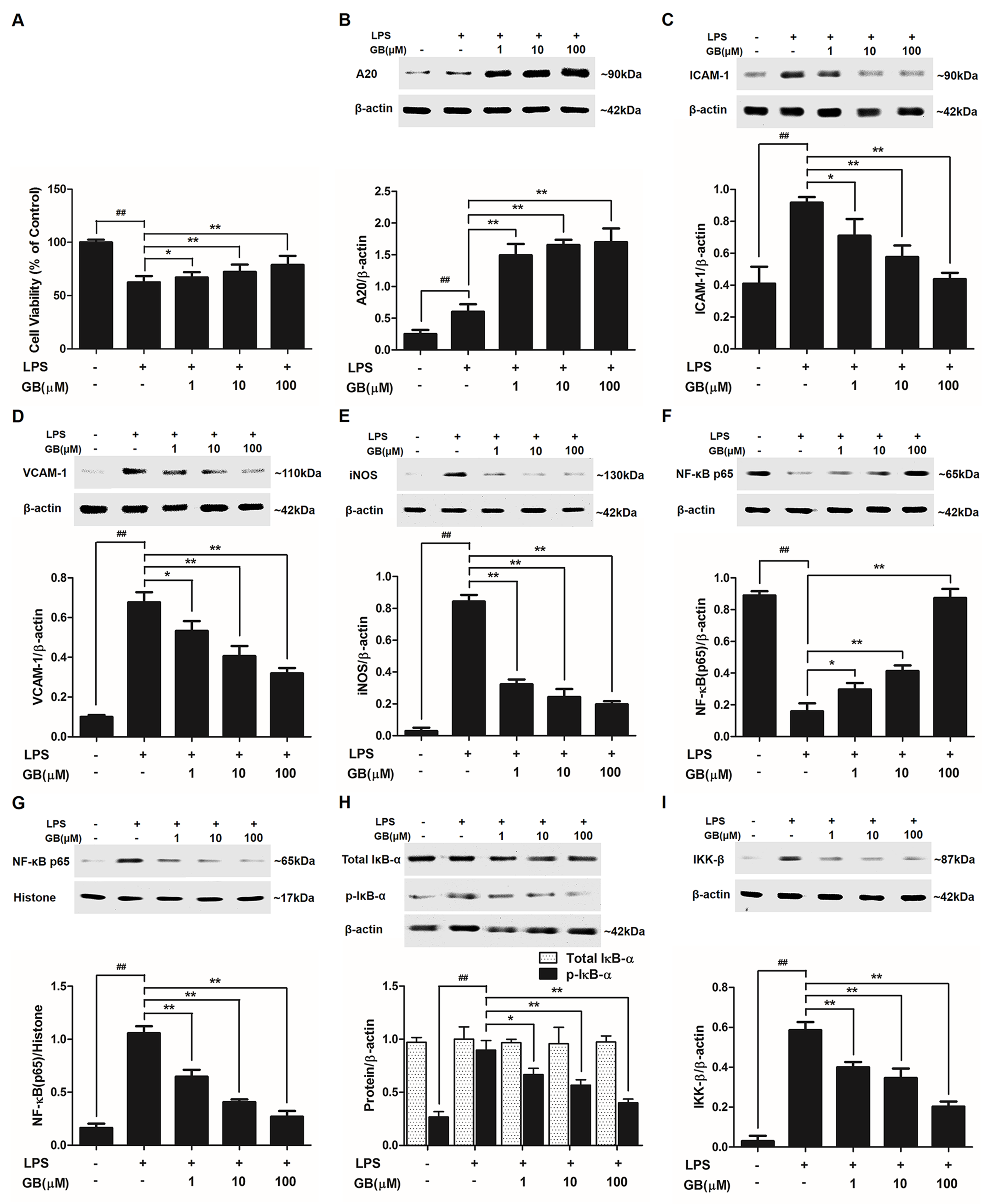

Supplement: Supplementary Figure 2 — Effects of GB on cell viability and the expressions of A20, ICAM-1, VCAM-1, iNOS, NF-κB p65, p-IκB-α, IKK-β after LPS procedure. [file Image_2.tif]

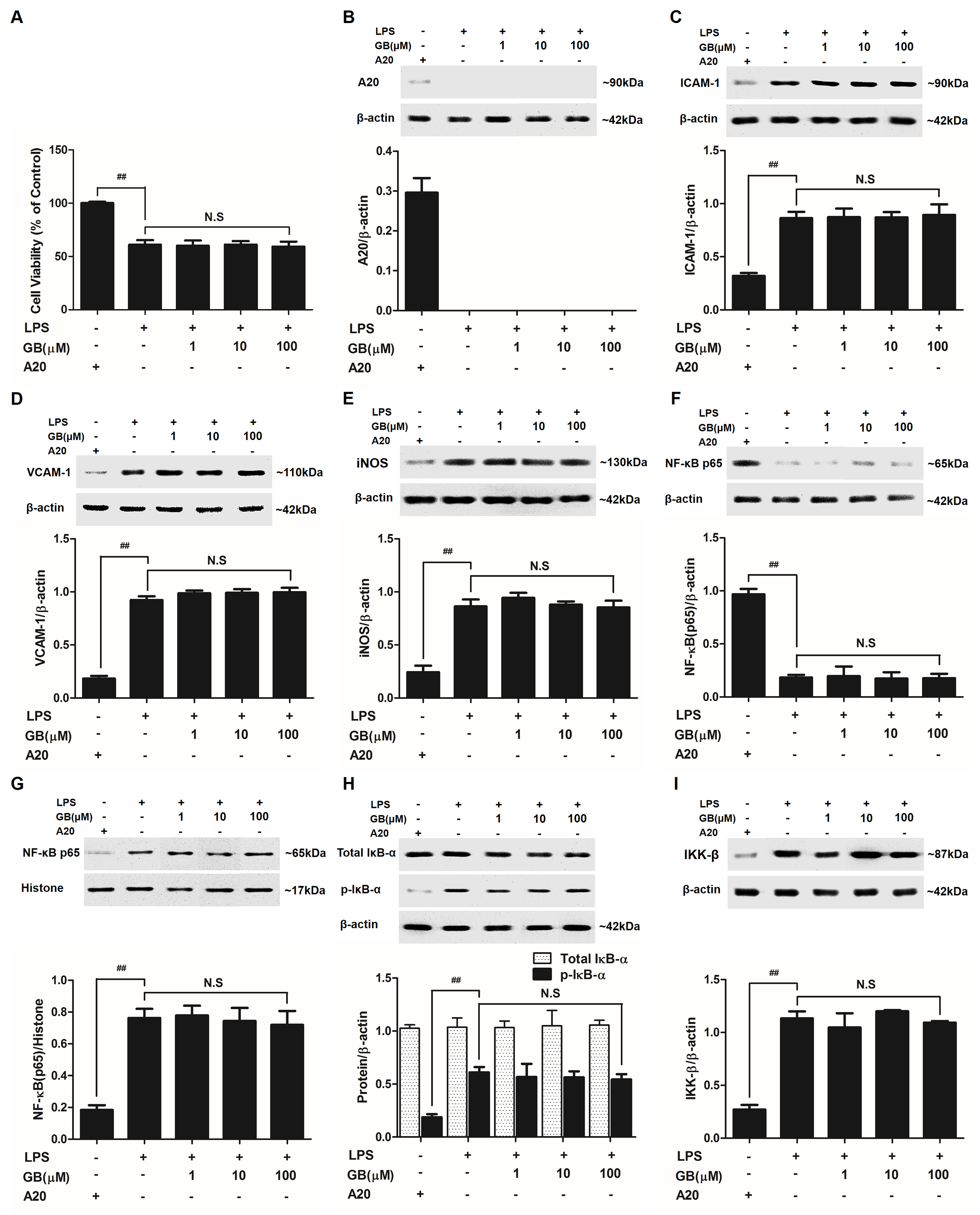

Supplement: Supplementary Figure 3 — Effects of GB on cell viability and the expressions of A20, ICAM-1, VCAM-1, iNOS, NF-κB p65, p-IκB-α, IKK-β after A20 silencing. [file Image_3.tif]
