# Supplementary material for: Cardioprotection of Ginkgolide B on Myocardial Ischemia/Reperfusion-Induced Inflammatory Injury via Regulation of A20-NF-κB Pathway
Source: Front Immunol. 2018 Dec 12;9:2844. doi: 10.3389/fimmu.2018.02844 (PMC6299132; doi:10.3389/fimmu.2018.02844)
Supplement: Supplementary file 4 [file Data_Sheet_1.docx]

**Materials and Methods**

***In vitro* LPS models in ventricular myocytes**

Rat ventricular myocytes were separated from the hearts of 1-4-day-old Sprague-Dawley rats according to trypsin enzymic digestion and differential attachment methods. Three days later, the cells were finally purified at a density of 1 × 10^5^/mL in DMEM medium supplemented with 10% fetal calf serum in 95% air/5% CO_2_ at 37°C.

LPS stimulation procedure (supplementary Fig. 1). The cells were incubated with LPS (2 μg/mL) for 24 h. Randomly selected cells were divided into 5 groups as follows (n = 8 per group): (1) Control group, cells were cultured in DMEM medium; (2) LPS group, cells were treated with 2 μg/mL LPS for 24 h; (3) 1 µM GB group, LPS cells were preincubated with 1 µM GB for 24 h; (4) 10 µM GB group, LPS cells were preincubated with 10 µM GB for 24 h; (5) 100 µM GB group, LPS cells were preincubated with 100 µM GB for 24 h.
