# Supplementary material for: Cardioprotection of Ginkgolide B on Myocardial Ischemia/Reperfusion-Induced Inflammatory Injury via Regulation of A20-NF-κB Pathway
Source: Front Immunol. 2018 Dec 12;9:2844. doi: 10.3389/fimmu.2018.02844 (PMC6299132; doi:10.3389/fimmu.2018.02844)
Supplement: Supplementary file 5 [file Data_Sheet_2.docx]

**Results**

**1. Effect of GB on LPS-induced inflammatory injury in ventricular myocytes**

**1.1 GB protected ventricular myocytes against LPS stimulation.** As shown in supplementary Fig. 2A, pre-treatment with LPS led to an obviously decrease to 62.5 ± 5.8% (P<0.01) in cell viability compared with control group. The data indicated that GB at the concentrations of 1, 10, and 100 µM could significantly increase the cell viability to 68.4 ± 2.3%, 72.4 ± 6.8%, and 78.8 ± 8.6% (P<0.01), respectively, compared with LPS group.

**1.2 GB increased the expression of A20 after LPS stimulation in ventricular myocytes.** As shown in supplementary Fig. 2B, the level of A20 was higher in LPS group (P<0.01 vs. control group). Moreover, 1, 10, 100 μM GB significantly increased A20 levels in response to LPS injury (P<0.01 vs. LPS group).

**1.3 GB inhibited overproduction of inflammatory cytokines after LPS stimulation in ventricular myocytes.** After LPS stimulation, the levels of TNF-α, IL-1β and IL-6 in the LPS group were significantly elevated by 12.34-fold, 14.55-fold and 42.92-fold (P<0.01) compared with control group (supplementary table 1). 1, 10, 100 µM GB significantly reduced the levels of TNF-α by 37.8%, 50.2% and 67.0% (P<0.01), IL-1β by 38.2%, 59.2% and 71.9% (P<0.01) and IL-6 by 41.3%, 65.8% and 86.6% (P<0.01) compared with LPS group.

**1.4 GB prevented overexpressions of ICAM-1, VCAM-1 and iNOS after LPS stimulation in ventricular myocytes.** The expressions of ICAM-1, VCAM-1 and iNOS in ventricular myocytes markedly increased to about 2.23-fold, 6.77-fold and 28.11-fold (P<0.01) after LPS stimulation, compared with control group (supplementary Fig. 2C, 2D and 2E). While, 1, 10, 100 μM GB could significantly reduce the expressions of ICAM-1 by 22.5%, 37.1% and 52.4% (P<0.01), VCAM-1 by 21.2% (P<0.05), 39.9% (P<0.01) and 52.7% (P<0.01) and iNOS by 61.7%, 71.1% and 76.7% (P<0.01) compared with LPS group.

**1.5 GB inhibited NF-κB p65 translocation after LPS stimulation in ventricular myocytes.** Supplementary Fig. 2F and 2G showed stimulation of cell with LPS could promote the translocation of NF-κB p65 from cytoplasm to the nucleus. Nonetheless, when pretreated with GB (1, 10, 100 μM) prior to induction with LPS, the level of p65 in cytoplasm did not decrease and there was no concomitant increase in nucleus.

**1.6 GB inhibited phosphorylation of IκB-α and activity of IKK-β after LPS stimulation in ventricular myocytes.** As shown in supplementary Fig. 2H and 2I, the expression of p-IκB-α in LPS group significantly increased by 3.36-fold (P<0.01 vs. control group). However, 1, 10, 100 μM GB all reduced the levels of p-IκB-α by 25.7%, 36.8% and 55.4% (P<0.01) compared with LPS group.

**2. Effect of GB on LPS-induced inflammatory injury in the presence of A20 gene silence in ventricular myocytes**

**2.1 GB could not increase cell viability after A20 gene silencing.** The cell viabilities in LPS + A20 silence group (supplementary Fig. 3A) were significantly reduced (P<0.01 vs. control group). After A20 gene silencing, GB could not elevate the cell viability against to LPS injury.

**2.2** **Effective and stable A20 gene silence in ventricular myocytes.** In control group, the ventricular myocytes were preincubated with pGPU6/Hygro and little A20 expressed after transfection. In addition, the ventricular myocytes in other groups were preincubated with pGPU6/Hygro-A20 and no A20 expressed after transfection (supplementary Fig. 3B).

**2.3 GB could not inhibit the expression of inflammatory factors after A20 gene silencing.** Compared with control group, the expressions of TNF-α, IL-1β, IL-6, ICAM-1, VCAM-1 and iNOS in LPS + A20 silence group were obviously increased. Whereas, after A20 gene silencing, GB could not influence the expressions of TNF-α, IL-1β, IL-6, ICAM-1, VCAM-1 and iNOS (supplementary table 2 and supplementary Fig. 3C, 3D, 3E, 3C, 3D, 3E).

**2.4 GB had no influence on translocation of NF-κB p65, phosphorylation of IκB-α and activity of IKK-β after A20 gene silencing.** The levels of NF-κB translocation, IκB-α phosphorylation and IKK-β activity were significantly affected in LPS + A20 silence group. Nevertheless, all GB groups had no impact on NF-κB p65 translocation, IκB-α phosphorylation and IKK-β activity compared with LPS + A20 silence group (shown in supplementary Fig. 3F, 3G, 3H and 3I).
