# Supplementary material for: Cardioprotection of Ginkgolide B on Myocardial Ischemia/Reperfusion-Induced Inflammatory Injury via Regulation of A20-NF-κB Pathway
Source: Front Immunol. 2018 Dec 12;9:2844. doi: 10.3389/fimmu.2018.02844 (PMC6299132; doi:10.3389/fimmu.2018.02844)
Supplement: Supplementary file 6 [file Table_1.docx]

**Supplementary table 1 Effects of GB on supernatant inflammatory cytokines of ventricular myocytes after LPS stimulation.**

| **Group** | **Concentration (µM)** | **TNF-α (pg/mL)** | **IL-1β (pg/mL)** | **IL-6 (pg/mL)** |
| --- | --- | --- | --- | --- |
| Control |  | 8.12±3.19 | 90.01±19.88 | 19.16±2.58 |
| LPS |  | 100.21±12.39^##^ | 1309.87±89.31^##^ | 822.29±32.19^##^ |
| LPS+GB | 1 | 62.29±8.36^**^ | 809.13±62.19^**^ | 483.01±36.60^**^ |
|  | 10 | 49.90±6.39^**^ | 533.98±48.10^**^ | 281.03±29.19^**^ |
|  | 100 | 33.02±4.83^**^ | 368.19±29.66^**^ | 110.15±32.55^**^ |

Values were expressed as mean ± SD (n = 8).

^##^P < 0.01 vs. control group; ^∗^P < 0.05, ^∗∗^P < 0.01 vs. LPS group.

**Supplementary table 2 Effects of GB on supernatant inflammatory cytokines of A20 silent ventricular myocytes after LPS stimulation.**

| **Group** | **Concentration (µM)** | **TNF-α (pg/mL)** | **IL-1β (pg/mL)** | **IL-6 (pg/mL)** |
| --- | --- | --- | --- | --- |
| Control |  | 10.13±2.37 | 86.36±7.16 | 22.19±3.63 |
| LPS+A20- |  | 103.19±7.11^##^ | 1209.84±100.32^##^ | 903.17±86.55^##^ |
| LPS+GB+A20- | 1 | 106.87±12.16 | 1309.29±120.98 | 879.39±38.21 |
|  | 10 | 98.37±8.09 | 1293.72±98.73 | 893.90±66.28 |
|  | 100 | 100.98±12.58 | 1200.84±100.33 | 853.22±79.36 |

Values were expressed as mean ± SD (n = 8).

^##^P < 0.01 vs. control group.
